# A Comparison of Three Types of “Vineyard Management” and Their Effects on the Structure of *Plasmopara viticola* Populations and Epidemic Dynamics of Grape Downy Mildew

**DOI:** 10.3390/plants11162175

**Published:** 2022-08-21

**Authors:** Shuyi Yu, Baihong Li, Tianshu Guan, Li Liu, Hui Wang, Changyuan Liu, Chaoqun Zang, Yuqian Huang, Chunhao Liang

**Affiliations:** 1 Institute of Plant Protection, Liaoning Academy of Agricultural Sciences, Shenyang 110161, China; 2 Plant Protection College, Shenyang Agricultural University, Shenyang 110866, China

**Keywords:** genetic structure, SSR, *Plasmopara viticola*, rain shelter, temporal dynamics

## Abstract

Grape downy mildew (GDM) is a destructive grapevine disease caused by *Plasmopara viticola* that occurs worldwide. In this study, we determined the characteristics of GDM epidemics and the grapevine canopy micro-climate in open-field, fungicide-spray, and rain-shelter plots during two constitutive years (2016 and 2017). It was found that rain shelter can significantly delay the disease occurrence by 28 and 21 days, reduce the epidemic phase by 28 and 21 days, and decrease the final disease index by 82% and 83%. Furthermore, it can block precipitation, reduce the relative humidity by 11% and 8%, and reduce the leaf wetness duration by 85% and 76% compared with open-field cultivation. A total of 3861, 783, and 1145 lesions were collected from the open-field, fungicide-managed, and rain-shelter plots, respectively, for analyses of the genetic diversity, population differentiation, and epidemic mode with seven microsatellite markers. In terms of genetic diversity, the Nei’s diversity index ranged from 0.569 to 0.680 and Shannon’s information index ranged from 0.958 to 1.226, showing high levels of diversity across populations. Similar to fungicide management, a rain shelter can significantly reduce the population’s genetic diversity. Low pairwise *FST* values (0.003–0.047) and high gene flow (*Nm* = 1.548–20.699) were observed among the three populations each year. In addition, most of the genetic variation occurred within populations. The epidemic mode of GDM in the open-field, fungicide-managed, and rain-shelter cultivation showed moderate, low, and high levels of clonality, respectively, in the case study.

## 1. Introduction

Grape downy mildew (GDM) is one of the most destructive diseases of grapevine (*Vitis vinifera* L.) worldwide. The pathogen of this disease, *Plasmopara viticola*, is a diploid obligate biotrophic oomycete [1]. This disease originated in wild *Vitis* species in North America [2], and it was accidentally introduced into European grape vineyards in the 1870s by native American rootstocks that were resistant to *Phylloxera* [3]. Since then, GDM has spread rapidly in global grape-growing areas, particularly those that are warm and humid [4,5]. *Plasmopara viticola* can reproduce sexually by producing thick-walled resistant oospores as well as asexually through the production of sporangia during the growing season [6]. Mature oospores that overwinter in infected leaf residue germinate in the early spring to produce sporangia, which release zoospores that cause the primary infections. The primary lesions produce abundant sporangia that are blown by the wind to cause secondary infections when the leaves are wet. The pathogen has a considerable number of asexual cycles during the season under suitable environmental conditions. GDM can damage all of the green tissues of grapevines, and the infection of leaves leads to a decrease in photosynthetic efficiency, which induces a decline in grape quality. Damages to the inflorescences and berries lead to direct yield losses [7]. Currently, the predominant strategy for controlling GDM is based on fungicide treatments [8]. Massive and repeated chemical applications throughout the grapevine growing seasons lead to many problems, such as high expenses for producing grapes, threats to human health, environmental contamination, the loss of GDM-resistant grape varieties, and fungicide resistance. Therefore, it is imperative to investigate new ecological strategies to control downy mildew and, simultaneously, to avoid negative effects on the environment and human health [9].

Liaoning Province is one of the primary grape-producing provinces in China, and it has the largest area for grapevines in the country and produces the highest yields of table grapes. However, hot and rainy conditions from spring to autumn in most areas of Liaoning Province coincide with the grape-growing season, which results in severe outbreaks and epidemics of GDM [10]. A rain shelter is a simple cultural practice of using a bracket composed of bamboo and wood or galvanized steel pipes, and a membrane made of polyethylene film to cover the top of the grapevine canopy [11]. The use of this technique to effectively inhibit the incidence of GDM, to improve fruit quality, and to increase grape yields was first confirmed in several east Asian countries [10,12,13]. Some vineyards in Liaoning Province have been using rain shelters to control GDM for more than a decade. Previous studies have shown that, at the end of the growing season, the disease index decreased by nearly 80% using this method compared with the growth of grapevines in an open field [14]. Little is known about the effects of different types of vineyard management on the temporal dynamics of GDM, the epidemic mode of the pathogen, and the contribution of sexual and asexual reproduction to GDM epidemics.

A study of the population genetics of *P. viticola* using microsatellite markers can help to better understand the quantitative contribution of distinct genotypes to GDM epidemics and the evolutionary genetics among different geographical populations. GDM epidemics are usually initiated by oospores in most regions of the world, including Europe, the USA, and China, which can overlap throughout the grape-growing seasons [15,16,17,18,19,20,21,22]. In some warm and isolated regions, such as the Greek islands, Australia, and South Africa, GDM epidemics are driven by asexual reproduction by sporangia during most of the growing season [23,24,25]. In addition, the populations of *P. viticola* in Australia, South Africa, and South America are more closely related to those in Europe compared with those in North America. The use of multiple gene genealogies has shown that the current Chinese *P. viticola* population is a combination of endemic strains and those introduced from the USA [26]. When rain shelters are introduced to address GDM epidemics, it remains unknown whether the epidemic characteristics of the disease or the genetic structure of the *P. viticola* population change. Taking into consideration the large area of cultivated grapevines in Liaoning Province and the many rain shelter vines that grow in proximity to the open fields, this study had three objectives: (1) to characterize the seasonal epidemics of GDM and canopy micro-environment between rain shelters and open-field systems with or without fungicide sprays; (2) to determine whether the three types of vineyard management systems, including open fields, fungicide sprays, and rain shelters, influence the *P. viticola* population structure; and (3) to perform a comparative temporal analysis of the *P. viticola* population genetic structure that affects populations in the three treatment environments.

## 2. Results

### 2.1. Disease Epidemic and Meteorological Conditions

**Figure 1:**
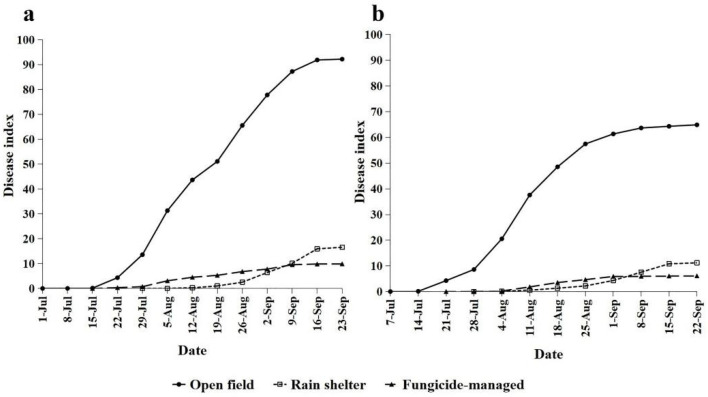
Temporal dynamics of the disease index of grape downy mildew (GDM) under open-field, rain-shelter, and fungicide-managed plots in Shenyang 2016 (**a**) and 2017 (**b**).

There were significant differences in the effects of the three management methods for the GDM epidemic. The first visibility of GDM in the open field appeared at approximately the same time (beginning of July) in 2016 and 2017 (Table 1). The GDM incidence then rose rapidly from mid-July to mid-August, and the disease index increased slowly from late August to mid-September (Figure 1). The development of GDM in 2016 was more favorable than during 2017 since the accumulated rainfall from July to September 2016 (428.6 mm) was much higher than that during the same period in 2017 (290.3 mm) (Table 2). In 2016 and 2017, the use of a rain shelter significantly delayed the initiation of epidemics by 28 days and the final disease index by 80% compared with the open-field treatments. In addition, the average apparent infection rate decreased to 0.114 in 2016 and to 0.106 in 2017, showing a difference from the open-field treatments (Table 1). A rain-shelter treatment can change the canopy micro-environment and can significantly reduce the relative humidity of the canopy and the duration of average daily leaf wetness. In particular, it sharply reduced precipitation. However, it had little effect on the canopy daily temperature (Table 2). Compared with the open-field treatments, the initiation of epidemics occurred after 14 days in the fungicide-managed plot, and the disease progressed at a slow rate throughout the growing seasons (0.089 in 2016 and 0.079 in 2017). The final disease index decreased by approximately 90% (Table 1). Considering that there was no significant difference in canopy microclimate between the spraying and open-field plots, the enormous difference in disease index between the two treatments was most likely due to the stringent spray programs that were applied.

### 2.2. Genotype Identification and Epidemic Mode of P. viticola

In 2016, 2110 genotypes were discriminated among 2314 lesions collected in the open-field treatment. The percentage of lesions caused by the dominant genotype (G_1_), second predominant genotype (G_2_), third predominant genotype (G_3_), single genotypes, and other genotypes were 23.7%, 10.5%, 6.3%, 39.7%, and 19.8%, respectively (Table 3). The open-field treatment was characterized by an intermediate level of clonality of 39.7% in 2016, according to the proportion of primary infection caused by oospores (total lesions caused by single genotypes) [27]. In contrast, the contribution of secondary infections caused by sporangia in the epidemics (sum of the lesions caused by predominant genotypes and other genotypes) was 60.3%. The three genotypes appeared >50 times in each investigation period. This included lesions from the same and adjacent diseased vines. In the conventional fungicide-managed plot, the collected lesions were much less than those in the open field plot with 423 genotypes detected. The majority of the genotypes detected were single genotypes, accounting for 329 (77.8%), which is >50%, indicating that the primary infection was the main driving force of the epidemic. Thus, the epidemic mode of *P. viticola* in the conventional fungicide-managed plot had a low level of clonality (Table 3). The population in the rain-shelter plot was significantly different from those of the other two treatments. The contribution of primary infection to the epidemic was only 7.9%. Therefore, the epidemic mode of *P. viticola* in this treatment had a high level of clonality, specifically in contrast to the conventional fungicide-managed plot (Table 3). 

In 2017, the total number of genotypes that were discriminated in the open-field treatment was 1369 (out of 1547 lesions). The percentage of lesions in this treatment caused by G_1_, G_2_, G_3_, single genotypes, and other genotypes were 21.6%, 10.0%, 4.7%, 41.1%, and 22.6%, respectively (Table 3). The proportion of primary and secondary infections in the epidemics was 41.1% and 58.9%, respectively. Accordingly, the 2017 epidemic mode of *P. viticola* in the open field had been medium, and 275 different genotypes were detected in the fungicide-managed plot. Most of the genotypes detected were the single genotypes that accounted for 229 (83.3%). Thus, there was a low epidemic mode of *P. viticola* in the fungicide-managed plot (Table 3). This was most likely owing to the stringent fungicide spraying program used to effectively inhibit the oospore replication from the whole growing season based on the data for two consecutive years. The contribution of primary infection to the epidemic was only 8.8%. Therefore, the epidemic mode of *P. viticola* was high in the rain-shelter plot (Table 3). 

The SSR analysis indicated that the same genotypes were not detected in both years, whereas the predominant genotypes were shared among the different treatment plots for each season. The three genotypes were consistent under the three treatments in each year. The dominant genotype (G_1_) was detected first in early July in the open-field plot. The genotype then generated progeny on the same or nearby vines. From mid-July to late July, G_1_ reproduced at only low frequencies in the fungicide-managed plots. G_1_ occurred two weeks later in the rain shelter than in the fungicide-managed plot. Owing to effective fungicide control and the surging lesions on both sides of the shelter, the lesions in the rain shelter exceeded those of the fungicide-managed plot by the beginning of September. The temporal dynamics of the G_2_ and G_3_ populations were the same as those of G_1_. However, the two genotypes were much less able to increase compared with G_1_ (Table 3).

### 2.3. Genetic Structure and Population Differentiation

The number of alleles (*A*) for each population varied from 3.286 to 4.143, the number of effective alleles (*Ae*) ranged from 2.542 to 3.390, Nei’s diversity index (*H*) ranged from 0.518 to 0.680, and Shannon’s information index (*I*) varied from 0.958 to 1.226 (Table 4). These four indices indicated that the open-field population had the highest genetic diversity, while the rain-shelter population showed the opposite or lowest genetic diversity. The populations in the open field had private alleles at the loci Pv14 (two) in 2016, and Pv7 (three) and Pv13 (one) in 2017. The populations in the fungicide-managed plots had private alleles at the loci Pv17 (two) in 2016, and Pv13 (one) and Pv17 (one) in 2017. However, no private alleles were detected in the isolates from rain shelter (Table 4). All of the populations revealed high genetic diversity in the three types of management, while both the fungicide and rain-shelter treatments reduced the genetic diversity of the *P. viticola* populations. A total of 51 alleles were detected among the six populations using the seven SSR markers generated among the polymorphic loci. The number of alleles per locus ranged from two (Pv16 and Pv39) to eight (Pv7). The most diverse locus was Pv7 (k = 8), followed by Pv17 (k = 7) (Table 4). Positive *Fis* values (0.02–0.79) were observed for most of the loci in each population, which indicates a heterozygous deficiency attributed to non-random mating.

Significant *Fst* values (*p* < 0.05) were observed among the populations of the open-field, fungicide-managed, and rain shelter treatments in the same year. However, there was no significant differentiation in the populations between sampling years in the three types of management. The pairwise population *Nm* values based on the *Fst* values were calculated to measure the gene flow between populations. High *Nm* values ranged from 5.159 to 20.699 in 2016 and from 1.548 to 6.221 in 2017 based on pairwise comparisons of the populations under the open-field, fungicide-managed, and rain-shelter treatments, implying that there was frequent gene exchange among the different populations in the same growing season (Table 5). A comparative analysis indicated that the *Das* of the seven alleles was consistent among the populations of the three treatments in both years (Table 6). However, Pv7 and Pv17 differed by only 1–4 bp in the fungicide-managed plot in 2017. The predominant genotypes in 2017 were derived from the variation in predominant genotypes in 2016. An AMOVA was used to partition the total genetic diversity among and within the populations (Table 7).

## 3. Discussion

In this study, the temporal dynamic parameters of GDM can be significantly affected in a rain-shelter treatment in northeast China, including a delay in the onset of the epidemic by more than 20 days, a reduction in the epidemic phase by more than 60 days, and a decrease in the final disease index by 80%. The ability of this viticultural mode to control GDM was similar to that of chemical applications, and it can be applied in warm and moist regions areas during the grape-growing season. A rain shelter can change the canopy climate, such as by blocking precipitation and by reducing the average daily relative humidity and the average duration of daily leaf wetness, compared with open-field treatments. The whole infection cycle of GDM, including the formation, maturation, germination, and release of oospores and sporangia, was severely inhibited by the lack of rain [27,28]. Previous studies have confirmed that rain shelters can reduce the severity of grape diseases effectively, such as grape ripe rot, white rot, downy mildew, grey mold, and brown spots [13]. A rain shelter can greatly reduce the frequency of chemical applications and the cost of growing in open-field viticulture management [11]. Due to the high temperature, low humidity, and reduced irradiation of the grape canopy micro-environment shaded by the rain shelter, the occurrence of grape powdery mildew was more serious than that in open-field cultivation. However, powdery mildew can be effectively controlled by using fungicides in the shelters [13]. A rain shelter effectively protects the berries from rot and drop and delays the maturation of fruits, which leads to an increase in grape yield [11,13]. In addition, the total sugar content, soluble solid content, and pH values of the mature berries from rain-sheltered plots were significantly higher than those from the fungicide-spray and non-treated plots, which improved the quality of berries [12,13].

All of the *P. viticola* populations in the open-field, fungicide-managed, and rain-shelter treatments were highly genetically diverse, which is consistent with previous findings in many countries [15,22,24]. We also found that the number of alleles in all of the loci of the open-field population was similar to that in the populations from Europe [19]. Gametic disequilibrium can be generated by several processes, including linkage, population admixture, genetic drift, population expansion, non-random mating, and selection [29]. Positive *Fis* values for five of the seven loci usually indicated that all of the populations deviated from the Hardy–Weinberg equilibrium owing to non-random mating. As an important base for breeding new table grape cultivars in China, Liaoning Province introduced a large number of new parental and nursery materials throughout the world over the past 30 years. Simultaneously, Liaoning Province is also a grapevine nursery region where a large number of cuttings, including new cultivars, are transferred to other grape-planting regions of China each year. The widespread distribution of *P. viticola* throughout China is due to the human-mediated dispersal of seedlings and grape cultivars [16,17]. Population genetic analyses showed that *P. viticola* populations among the open-field, fungicide-managed, and rain-shelter treatments in two consecutive years belong to the same metapopulation because there is significant low population differentiation among the three types of vineyard management in each year (*Fst* = 0.003 *–0.012 * in 2016 and *Fst* = 0.013 *–0.047 * in 2017) and predominant genotypes (G_1_, G_2_, and G_3_) were shared each year among three populations, which provides direct evidence for effective genotype flow among treatments. Nonetheless, it is important to note that the predominant genotype in the open-field cultivation was not the predominant genotype in the fungicide-treated cultivation, but that was the absolute predominant genotype in the rain-shelter cultivation. It was confirmed that fungicide management and rain shelters significantly changed the genetic diversity of the *P. viticola* populations. The loss of alleles and lower private allelic richness at mutation-drift disequilibrium in the rain-shelter plots, compared with the open-field and fungicide-application plots, are typical signatures of genetic bottlenecks [17]. Moreover, high levels of gene flow among the populations suggest that immigration is another main reason for the deviation from the Hardy–Weinberg equilibrium. Based on the analysis of molecular variation, it can be further confirmed that the genetic variation in populations primarily originates within the population rather than between populations, reflecting the frequent gene exchange between populations and the low degree of genetic variation [30].

The genetic structure of *P. viticola* populations was identified by genotyping 3861 lesions from the open-field plots, 783 lesions from the fungicide-managed plots, and 1145 lesions from the rain-shelter plots with the seven SSR markers across both years. Previous results showed that both oospores and sporangial infections promote overlapping GDM epidemics during the entire grape-growing season [17,20,24,28,31]. The epidemics were characterized by an intermediate level of clonality in the open-field plots, which was similar to previously reported populations in some European vineyards [16,31]. However, we found that the populations of the fungicide-managed plots contained a low level of clonality, which is consistent with previous reports on the Chinese populations [22]. Both genotypic diversity and population numbers were significantly reduced under the pressures of fungicides. Some of the reasons for the low level of clonality include unfavorable environmental conditions, such as high temperatures [28], low humidity [6], long-wavelength UV [32], and fungicide applications during the growing period [1]. These factors can significantly affect the capacity for asexual infection, such as sporangiophore formation, sporangium dissemination, and germination. However, oospores are strongly resistant to adverse environmental conditions and chemicals, and they germinate continuously, which causes new infections throughout each growing season [33]. Thus, we hypothesize that oospore infections (sexual infection) play a dominant role in epidemics in northeast China, particularly in fungicide-managed regions. It is similar to those of most countries worldwide. In contrast, the GDM epidemic mode in the rain shelter showed a high level of clonality. The main explanation for this could be the change in the canopy micro-environment caused by the rain shelters, including a lower duration of leaf wetness and relative humidity. The canopy environment becomes unsuitable for the lifecycle of the pathogen, and only the vines around both sides of the shelter can be effectively infected. Similarly, in western Australian, South African, and Greek vineyards, a proportion of the oospore-derived lesions decreased rapidly to low values (1% to 44%) following the seasonal onset of the disease [23,24,31,34]. Therefore, both specific ecological environments (warm climate and geographical isolation) and human intervention (fungicide applications and rain shelter) significantly changed the genetic structure of the *P. viticola* populations. Many prediction models of GDM in the open-field and fungicide-managed cultivations mainly depend on favorable conditions for oospore germination, but such models cannot be accurately applied under rain shelter cultivations. Therefore, new models should be developed based on suitable conditions for the survival and infection of asexual spores under rain-shelter cultivation.

We divided the genotypes into three groups based on their clone frequency during epidemics. These included predominant genotypes (G_1_, G_2_, and G_3_), single genotypes, and other genotypes. The accumulation of the three genotype groups in different treatments was consistent with the temporal dynamics of GDM in both years. Most clonal infections were caused by a few predominant genotypes. This is the epidemic pattern of the natural disease. In general, only two or three genotypes played a principal role in the epidemics in both years, replicating at least 50 times throughout the season. These genotypes had two advantages. On the one hand, they occurred early and usually at the center of the plot, which provided greater opportunities to rapidly expand [3]. Alternatively, the genotypes had a genetic advantage allowing them to dominate the epidemics by elevating the heterozygosity and chromosome numbers of *P. viticola* [24,31]. The ability of most of the progeny of specific genotypes to reproduce asexually following sexual infection is limited. Only the ‘other genotype’ successfully infected new vines around the vine of the first appearance of this clone, reproducing less than 10 times and lasting for about a month; this was probably because the sporangia are highly sensitive to unfavorable environmental conditions [33,35]. Oospores played a key role in the primary infection of GDM and released new genotypes to ensure the development of epidemics throughout the growing season. The formation and maturation of oospores drives the high genetic variation in GDM, which affects the adaptability of the pathogen to external conditions, including the environment, varietal resistance, resistance to fungicides, and others [36]. None of the genotypes were able to spread across the years in this study. This suggests that *P. viticola* only overwinters as oospores in northeast China but not as mycelia or sporangia.

## 4. Materials and Methods

### 4.1. Experimental Vineyard 

The experiments were conducted during two consecutive growing seasons from 2016 to 2017 in one naturally infected vineyard. The vineyard is situated in Shenyang, Liaoning Province, China (41°49′13″ N 123°32′16″ W, at 50 m a.s.l.). The cultivar was Centennial Seedless, which is highly susceptible to GDM. Three treatment patterns were implemented in a parallel setting that included management by fungicides, open fields, and rain shelters that were located from the west to east sides of the vineyard. Each experimental plot contained eight rows with 50 vines each. The vines were spaced 1.0 m apart within rows and 1.5 m apart between rows in each treatment pattern. The vines were vertical trellis with cordon pruning at 1.5 m × 1.0 m in each treatment pattern, the height of the trunk was generally 1.5 m when leaving the same number of buds per plant (12 buds/vine). The vines were oriented north–south on clayey soil and cultivated according to the local conventional management. The conventional plots managed with fungicides were alternately sprayed with commercial downy mildew fungicides, including 50% dimethomorph WP (BASF SE, Ludwigshafen, Germany) and 10% cyazofamid SC (Ishihara Sangyo Kaisha, Ltd., Osaka, Japan) approximately every two weeks from mid-June to early September. The rain shelter was built along with the vine rows and consisted of a steel trellis that was 11 m wide and 2.5 m high that was covered with clear and transparent polyethylene film from mid-May to early October during both years of the experiment [10,30]. The grapevines under the rain shelter were irrigated with drip irrigation, and the amount of irrigation was 6500 m^3^/hm^2^.

### 4.2. Meteorological Data Collection

Weather data, which included the average daily air temperature, relative humidity, rainfall, and leaf surface wetness duration, were collected using a TRM-ZS3 field microclimate observation station (Jinzhou Sunshine Technology Co. Ltd., Jinzhou, China) located in the middle of the vineyard. Temperature and relative humidity were determined from a portable micrologger (HOBO H8 Pro; Onset Computer Corp., Pocasset, MA, USA) on the canopy of each plot.

### 4.3. Disease Assessment

The experiments were conducted in naturally infected fields. The disease severity was assessed every 7 days from the first observation of the disease symptoms to the end of the grape-growing season each year. Thirty plants in each plot were randomly selected and tagged. The disease index of GDM was calculated using a 0–9 scale according to the GB/T 17980.122-2004 guidelines (Pesticide-Guidelines for the field efficacy trials (II)-Part 122: Fungicides against grape downy mildew) [37]. The disease index and average apparent infection rate were calculated using the following formulae:

(1) $$\mathrm{Disease}\;\mathrm{index}=\frac{\sum_{0}^{9}x_{i}y_{i}}{x_{\max}\sum y_{i}}\times 100$$

where *x_i_* is the value in each disease grade, *y_i_* is the number of diseased leaves in each disease grade, and *x_max_* is the highest value of the disease grade [38].

(2) $$\mathrm{Apparent}\;\mathrm{infection}\;\mathrm{rate}\;(r)=\frac{1}{t_{2}-t_{1}}(\ln\frac{x_{2}}{1-x_{2}}-\ln\frac{x_{1}}{1-x_{1}})$$

where *r* is the apparent infection rate between *t*_1_ and *t*_2_, *t*_1_ is the time at the first observation, *t*_2_ is the time at the second observation, *x*_1_ is the disease index at the first observation, and *x*_2_ is the disease index at the second observation [38]. Thirty vines per plot were used for data analysis, of which ten vines were used as one replicate, and a total of three replicates were set. The differences between downy mildew management were determined using a one-way analysis of variance (ANOVA) followed by Duncan’s new multiple range method at *p* = 0.05. SPSS 24.0 (IBM, Inc., Armonk, NY, USA) was used to perform these calculations.

### 4.4. Sampling

Lesions with GDM-like symptoms were collected at nearly all two-week intervals during the 2016 and 2017 grape-growing seasons. Each single lesion was collected by removing half of the lesion with scissors sterilized with 75% ethanol and by placing it in a 2 mL Eppendorf tube. The remainder of the lesion was left to ensure the survival of the genotype in the vineyard [16]. The locations of the sampled lesions were recorded based on the row and column number. The lesions from the same vine were considered to have been collected at the same coordinates in the plot. Two different sampling strategies were used to collect the lesions on each vine. The choice of total or partial sampling strategy (TSS or PSS, respectively) was determined by the disease severity. TSS was performed when the disease incidence was low (an average of <five lesions per vine), and all of the visible lesions could be collected. PSS was performed at a stage of high disease incidence (>five lesions per vine), and three single lesions were randomly sampled on each vine.

### 4.5. Sample Processing and Simple Sequence Repeats (SSR) Genotyping

High-throughput methods were used to extract the DNA from samples that were collected, as described by Gobbin et al. (2003). Seven polymorphic *P. viticola*-specific SSR markers (Pv7, Pv13, Pv14, Pv16, Pv17, Pv31, and Pv39) were amplified to genotype the collected samples [19,21]. A PCR reaction was performed for each SSR primer pair in a 15 μL total volume that contained 1–2 μL of genomic DNA (average 2.2 μg/μL), 1.5 μL of 10× PCR buffer, 0.45 μL of 25 mmol/L MgCl_2_ (Sangon Biotech, Shanghai, China), 0.2 μL of 10 mmol/L dNTPs (TIANGEN, Beijing, China), 0.5 μL of 5 U/μL Taq Polymerase (TIANGEN), and 0.2 μL of each 10 μmol/L forward and reverse primers (Sangon Biotech, Dalian, China). The PCR conditions were 94 °C for 5 min; 35 cycles at 95 °C for 30 s, 60 °C for 30 s, and 72 °C for 30 s; and a final elongation step at 72 °C for 10 min [19]. The PCR products were separated and identified using 8% polyacrylamide gel electrophoresis with 10× TBE buffer.

Genotypes were defined as unique SSR allele patterns following PCR amplification with the seven microsatellite markers. The classification of lesions in the genotypes that were analyzed was based on the assumption that lesions with the same allele pattern were considered clones of the same oospore, while the ones that presented a different allele pattern were interpreted as having been derived from independent oospores [15,17,24]. All of the genotypes could be divided into predominant genotypes that occurred at least 50 times in every single season, with single genotypes appearing once or other genotypes occurring < 50 times throughout the survey period. The genotypes with the three highest frequencies of occurrence were designated G_1_, G_2_, and G_3_ for the first, second, and third predominant genotypes, respectively. The contribution of genotypes to the GDM epidemic was described as the percentage of total number of lesions per genotype in a survey period (one investigation time or a single growing season). Primary infections that contributed to the GDM epidemic were defined as the sum of percentage of the total number of lesions of single genotypes. In contrast, secondary infections were identified as the percentage of the total number of lesions of predominant and other genotypes. Based on the proportion of sexual reproduction that contributed to the GDM infections, the epidemic was divided into three groups: low, intermediate, and high degrees of clonality, which were >50%, 33–50%, and <33% of the total single genotype lesions, respectively [27].

### 4.6. Genetic Analysis

The number of alleles per locus (*A*), effective alleles (*Ae*), private alleles (*Pa*), dominant allele size (*Das*), observed heterozygosity (*Ho*), expected heterozygosity (*He*), Nei’s gene diversity (*H*) [39], and Shannon’s information index (*I*) [40] within each population corrected for each clone for each locus were analyzed using GenALEx 6.5 [41] to evaluate their genetic diversity. The inbreeding coefficient (*Fis*) was determined within the populations corrected for clones with a 1000-permutations test using Genetix [42]. Positive *Fis* values generally indicate a reduction in heterozygosity, while negative *Fis* values usually indicate an increase in heterozygosity [43]. Population differentiation (*Fst*) and gene flow (*Nm*) between all the pairs of populations were measured using GenAlEx 6.5 [41] to estimate the genetic differentiation [44]. The significance of the *Fst* value was tested using 999 random permutations. An analysis of molecular variance (AMOVA) was performed to evaluate the genetic variation in GenAlEx 6.5.

## 5. Conclusions

Our current research shows that a rain-shelter cultivation can significantly affected the temporal dynamics of GDM by changing the canopy climate, especially precipitation, humidity, and the duration of daily leaf wetness. The control effect of this viticulture mode on GDM was similar to that of chemical applications, and it can be applied in warm and moist climates during the grape-growing season. *P*. *viticola* populations in open fields, fungicide sprays, and rain shelters were found to be distinct. The data obtained suggest that epidemiological and population genetic features of *P*. *viticola* may be further complicated in the future due to its capacity to adapt to different vineyard managements in many areas of the world.

## Figures and Tables

**Table 1:** Characteristics of grape downy mildew (GDM) under the open-field, rain-shelter, and fungicide-managed plots in Shenyang (2016–2017).

Treatment	First Visibility of the Disease		Epidemic Phase (d)		Disease Index		Average Apparent Infection Rate	
	2016	2017	2016	2017	2016	2017	2016	2017
Open field	7/1 a	7/7 a	92 a	86 a	92.22 a	64.89 a	0.121 a	0.112 a
Rain shelter	7/29 c	7/28 b	64 c	65 c	16.58 b	11.21 b	0.114 a	0.106 a
Fungicide	7/15 b	7/21 c	78 b	72 b	9.92 c	6.13 c	0.089 b	0.079 b

The first visibility of the disease indicates the date when the disease occurred; the epidemic phase indicates the period of disease incidence during the growing season; disease index indicates the mean value of the last disease investigation. Different letters indicate statistically significant differences among treatments (ANOVA followed by Duncan’s test; *p* < 0.05; *n* = 3).

**Table 2:** Changes in the canopy microclimate under open-field, rain-shelter, and fungicide-managed plots in Shenyang (2016–2017).

Treatment	Precipitation (mm)		Average Temperature (°C)		Average Relative Humidity (%)		Average Leaf Wetness Duration per Day (h)	
	2016	2017	2016	2017	2016	2017	2016	2017
Open field	428.6 a	290.3 a	23.4 a	23.3 a	67.7 a	62.1 a	2.7 a	1.7 a
Rain shelter	0 b	0 b	23.8 a	23.7 a	60.1 b	57.5 b	0.4 b	0.4 b
Fungicide	425.9 a	277.7 a	23.7 a	23.5 a	65.0 a	61.3 a	2.5 a	1.6 a

Canopy microclimate data were collected from the canopies of three vineyard managements using electronic sensors from 1 July to 30 September in 2016 and 2017. Different letters indicate statistically significant differences among vineyard managements (ANOVA followed by Duncan’s test; *p* < 0.05; *n* = 3).

**Table 3:** Sampling, lesions collected and analyzed, and number and temporal dynamic of *Plasmopara viticola* genotypes in open-field, fungicide-managed and rain-shelter cultivations during the period 2016–2017.

Cultivation Mode	Date	Collecting Method	Lesion Collected	Number of Genotypes	G_1_ (%)	G_2_ (%)	G_3_ (%)	Single Genotypes (%)	Other Genotypes (%)	*I*
2016 Open field	1 July	TSS	1	1	1	-	-	-	-	0.594
	15 July	TSS	316	281	31	9	5	144	92	0.967
	29 July	PSS	554	504	116	47	21	213	107	1.097
	12 August	PSS	1102	1019	273	126	84	387	149	1.270
	26 August	PSS	225	204	58	22	15	61	48	1.263
	9 September	PSS	116	101	22	18	7	33	21	1.243
	Total		2314	2110	501 (23.7)	222 (10.5)	132 (6.3)	838 (39.7)	417 (19.8)	1.226
2016 Fungicide	15 July	TSS	22	19	2	1	-	11	3	0.906
	29 July	TSS	78	70	5	3	2	54	6	1.079
	12 August	TSS	189	162	11	8	3	128	12	1.109
	26 August	TSS	125	116	8	4	1	95	8	1.265
	9 September	TSS	62	56	4	5	2	41	4	1.261
	Total		476	423	30 (7.1)	21 (5.0)	8 (1.9)	329 (77.8)	33 (7.8)	1.222
2016 Rain shelter	29 July	TSS	2	2	2	-	-	-	-	0.594
	12 August	TSS	72	66	25	9	6	12	14	1.101
	26 August	PSS	265	246	115	54	28	11	38	1.104
	9 September	PSS	264	233	102	36	24	17	54	1.124
	23 September	PSS	166	148	48	23	16	15	46	1.141
	Total		769	695	292 (42.0)	122 (17.6)	74 (10.6)	55 (7.9)	152 (21.9)	1.124
2017 Open field	7 July	TSS	1	1	1	-	-	-	-	0.297
	21 July	TSS	183	167	18	11	5	95	38	0.896
	4 August	PSS	636	524	113	49	27	219	116	1.060
	18 August	PSS	598	557	137	65	28	195	132	1.118
	1 September	PSS	129	120	27	12	4	53	24	1.121
	Total		1547	1369	296 (21.6)	137 (10.0)	64 (4.7)	562 (41.1)	310 (22.6)	1.093
2017 Fungicide	21 July	TSS	13	13	-	-	-	13	-	0.779
	4 August	TSS	32	27	2	-	-	25	-	0.907
	18 August	TSS	112	99	6	2	-	86	5	0.915
	1 September	TSS	98	88	11	5	2	67	3	0.996
	15 September	TSS	52	48	4	2	1	38	3	0.970
	Total		307	275	23 (8.4)	9 (3.3)	3 (1.1)	229 (83.3)	11 (4.0)	0.958
2017 Rain shelter	4 August	TSS	5	4	2	-	2	-	-	0.743
	18 August	TSS	42	37	12	8	3	5	9	0.940
	1 September	PSS	95	86	35	17	9	8	17	0.956
	15 September	PSS	168	153	59	32	21	12	29	0.954
	29 September	PSS	66	59	21	14	9	5	10	0.966
	Total		376	339	129 (38.1)	71 (20.9)	44 (13.0)	30 (8.8)	65 (19.2)	0.964

TSS, total sampling strategy; PSS, partial sampling strategy; G_1_, dominant genotype, followed by the percentage of lesions with the specific genotype in brackets; G_2_, the second dominant genotype; G_3_, the third dominant genotype; *I*, Shannon’s information index.

**Table 4:** Genetic diversity of *Plasmopara viticola* populations by seven microsatellite markers in open-field, fungicide-managed and rain-shelter cultivations during the period 2016–2017.

Population	Number of Alleles (k)							*A*	*A_e_*	*Pa*	*H*	*I*
	k_Pv7_	k_Pv13_	k_Pv14_	k_Pv16_	k_Pv17_	k_Pv31_	k_Pv39_					
2016 Open field	5	4	3	3	5	4	3	4.143	3.257	2	0.674	1.226
2016 Fungicide	5	3	3	3	7	4	3	4.000	3.390	2	0.680	1.122
2016 Rain shelter	4	4	3	2	5	4	3	3.571	2.995	0	0.640	1.224
2017 Open field	8	4	5	2	6	3	2	4.143	2.940	4	0.518	1.093
2017 Fungicide	3	3	3	2	5	3	2	3.286	2.578	2	0.518	0.958
2017 Rain shelter	3	3	5	2	5	3	2	3.286	2.542	0	0.444	0.964

*A*, number of alleles; *Ae*, number of effective alleles; *Pa*, number of private alleles; *H*, Nei’s gene diversity; *I*, Shannon’s information index.

**Table 5:** Estimation pair-wise *Fst* (below diagonal) and gene flow values (*Nm* = above diagonal) averaged over seven microsatellite loci of *Plasmopara viticola* populations in open-field, fungicide-managed, and rain-shelter cultivations during the period 2016–2017.

	2016 Open Field	2016 Fungicide	2016 Rain Shelter	2017 Open Field	2017 Fungicide	2017 Rain Shelter
2016 Open field	-	12.540	20.699	0.757	0.582	0.670
2016 Fungicide	0.005 *	-	5.159	0.740	0.570	0.654
2016 Rain shelter	0.003 *	0.012 *	-	0.776	0.530	0.660
2017 Open field	0.076 ns	0.083 ns	0.073	-	4.548	6.221
2017 Fungicide	0.097 ns	0.101 ns	0.096	0.016 *	-	1.548
2017 Rain shelter	0.089 ns	0.096 ns	0.083	0.013 *	0.047 *	-

* significant correlations at the 5% confidence level; ns, no significant correlations at the 0.05 level.

**Table 6:** The observed and expected heterozygosity (*H_o_* and *H_e_*), *Fis* and *Das* for each locus in *Plasmopara viticola* populations in open-field, fungicide-managed, and rain-shelter cultivations during the period 2016–2017.

Population	Pv7			Pv13			Pv14			Pv16			Pv17			Pv31			Pv39		
	*H_o_*/*H_e_*	*Fis*	*Das*	*H_o_*/*H_e_*	*Fis*	*Das*	*H_o_*/*H_e_*	*Fis*	*Das*	*H_o_*/*H_e_*	*Fis*	*Das*	*H_o_*/*H_e_*	*Fis*	*Das*	*H_o_*/*H_e_*	*Fis*	*Das*	*H_o_*/*H_e_*	*Fis*	*Das*
2016 Open field	0.76/0.79	0.05	291	0.72/0.76	0.05	218	0.33/0.49	0.32	122	0.68/0.66	−0.04	251	0.89/0.94	0.05	162	0.81/0.83	0.02	241	0.64/0.65	0.02	174
2016 Fungicide	0.77/0.72	−0.07	292	0.59/0.64	0.07	218	0.55/0.64	0.14	122	0.53/0.56	0.04	251	0.85/0.80	−0.07	166	0.71/0.75	0.05	241	0.50/0.56	0.11	174
2016 Rain shelter	0.84/0.89	0.05	291	0.71/0.76	0.06	218	0.27/0.48	0.45	122	0.75/0.48	−0.55	251	0.95/0.72	−0.33	162	0.90/0.92	0.02	241	0.70/0.73	0.04	174
2017 Open field	0.30/0.54	0.44	293	0.74/0.78	0.06	218	0.34/0.55	0.39	124	0.63/0.47	−0.34	251	0.47/0.62	0.24	168	0.75/0.77	0.02	245	0.27/0.34	0.20	174
2017 Fungicide	0.45/0.54	0.20	293	0.59/0.53	0.06	218	0.38/0.54	0.45	124	0.55/0.50	−0.55	251	0.65/0.77	−0.33	168	0.51/0.53	0.02	245	0.48/0.49	0.04	174
2017 Rain shelter	0.12/0.58	0.79	293	0.68/0.63	−0.08	218	0.33/0.66	0.50	124	0.70/0.48	−0.45	251	0.30/0.68	0.56	168	0.84/0.91	0.08	245	0.12/0.21	0.41	174

*Ho*, observed heterozygosity; *He*, expected heterozygosity; *Fis*, inbreeding coefficient; *Das*, dominant allele sizes.

**Table 7:** Analysis of molecular variance (AMOVA) for *Plasmopara viticola* populations in open-field, fungicide-managed, and rain shelter cultivations during the period 2016–2017.

Source of Variation	df	Sum of Squares	Estimate Variance	Percentage of Variation
Between years	1	4458.657	0.742	10.73
Among populations within years	2	808.672	0.230	4.07
Within Populations	5205	24,672.350	4.875	85.20
Total	5208	29,939.680	5.847	100.00

## Data Availability

Not applicable.
